# *Fusarium solani* Infection Depressed Photosystem Performance by Inducing Foliage Wilting in Apple Seedlings

**DOI:** 10.3389/fpls.2018.00479

**Published:** 2018-05-07

**Authors:** Kun Yan, Guangxuan Han, Chenggang Ren, Shijie Zhao, Xiaoqing Wu, Tiantian Bian

**Affiliations:** ^1^Key Laboratory of Coastal Environmental Processes and Ecological Remediation, Yantai Institute of Coastal Zone Research, Chinese Academy of Sciences, Yantai, China; ^2^Key Laboratory of Coastal Biology and Bioresource Utilization, Yantai Institute of Coastal Zone Research, Chinese Academy of Sciences, Yantai, China; ^3^State Key Laboratory of Crop Biology, Shandong Agricultural University, Tai’an, China; ^4^Shandong Provincial Key Laboratory of Applied Microbiology, Ecology Institute, Shandong Academy of Sciences, Jinan, China; ^5^School of Life Sciences, Ludong University, Yantai, China

**Keywords:** photosynthetic electron transport, photosystems interaction, plastoquinone, soil-borne pathogen, water deficit

## Abstract

*Fusarium* fungi are soil-borne pathogens, and the pathological effects on plant photosystems remain unclear. This study aimed to deeply reveal pathological characterization in apple seedlings infected with *Fusarium solani* by investigating photosystems performance and interaction. Roots were immersed in conidial suspension for inoculation. Thereafter, prompt and delayed chlorophyll *a* fluorescence and modulated 820 nm reflection were simultaneously detected. After 30 days of infection, leaf relative water content and dry weight were remarkably decreased by 55.7 and 47.1%, suggesting that the infected seedlings were subjected to *Fusarium*-induced water deficit stress. PSI reaction center was more susceptible than PSII reaction center in infected seedlings due to greater decrease in the maximal photochemical efficiency of PSI than that of PSII, but PSI reaction center injury was aggravated slowly, as PSII injury could partly protect PSI by restricting electron donation. PSII donor and acceptor sides were also damaged after 20 days of infection, and the restricted electron donation induced PSII and PSI disconnection by blocking PSI re-reduction. In accordance with greater damage of PSI reaction center, PSI oxidation was also suppressed. Notably, significantly increased efficiency of electron transport from plastoquinone (PQ) to PSI acceptors (REo/ETo) after 20 days of infection suggested greater inhibition on PQ reduction than re-oxidation, and the protection for PSI acceptors might alleviate the reduction of electron transport efficiency beyond PQ upon damaged PSI reaction center. Lowered delayed fluorescence in microsecond domain verified PSII damage in infected seedlings, and elevated delayed fluorescence in sub-millisecond domain during PQ reduction process conformed to increased REo/ETo. In conclusion, *F. solani* infection depressed PSII and PSI performance and destroyed their coordination by inducing pathological wilting in apple seedlings. It may be a pathogenic mechanism of *Fusarium* to induce plant photosystems damage.

## Introduction

Apple replant disease is a serious problem in major apple-growing regions in the world (Mazzola and Manici, 2012). In contrast to abiotic factors such as soil structure and nutrition, the primary origin inducing apple replant disease appears to be soil-borne pathogens, because this disease can be effectively prevented or alleviated by soil disinfection (Yim et al., 2013; Henfrey et al., 2015). To date, some fungi including *Fusarium, Rhizoctonia*, and *Cylindrocarpon* have been defined as the main soil-borne pathogens for apple replant disease (Tewoldemedhin et al., 2011a,b,c). Notably, considerable *Fusarium* fungi exist in replanted apple soil around Bohai Bay in China, and apple seedlings exhibit great susceptibility to these pathogens (Yin et al., 2017). Yantai city is an important apple planting area around Bohai bay in China. At present, about 50% orchards in Yantai are nearly 30 years old and should be timely reconstructed in these years. Accordingly, young apple trees will be in danger of replant disease in these reconstructed orchards. In fact, replant disease with reduced apple growth and yield have already appeared in some reconstructed orchards in Yantai. Many *Fusarium* fungi were also found in the soil of these orchards, and the most abundant species was *Fusarium solani*.

*Fusarium* fungi can invade plant vascular tissues, impede water transport through xylem by inducing vessel plugging, and lead to foliage wilt. Therefore, wilt symptoms and physiological responses in plants after *Fusarium* infection are similar to those upon water deficit stress (Wang et al., 2015). Photosynthesis is one of the most important metabolisms for plant growth and its sensitivity to *Fusarium* infection has been demonstrated in banana, tomato and wheat (Nogues et al., 2002; Dong et al., 2016; Yang et al., 2016). At early stage of *Fusarium* infection, stomatal limitation was mainly responsible for decreased CO_2_ assimilation because of stomatal closure under leaf water deficit (Nogues et al., 2002; Yang et al., 2016). The declined CO_2_ assimilation may induce oxidative stress in chloroplast with elevated generation of reactive oxygen species (ROS) by feedback inhibition of photosynthetic electron transport (Takahashi and Murata, 2008; Sonoike, 2011). As a consequence, carboxylation efficiency and photochemical capacity of PSII were reduced with aggravation of *Fusarium* infection, suggesting the negative effects on Rubisco and PSII reaction center (Pshibytko et al., 2006; Dong et al., 2016). Up to now, responses of photosystems performance to *Fusarium* infection have not been deeply explored compared with CO_2_ assimilation process in photosynthesis. Besides depressed photochemical capacity of PSII reaction center, electron transport capacity at PSII donor and acceptor sides remains to be elucidated in plants infected by *Fusarium*, and moreover, PSI performance and interaction between PSII and PSI are also largely unknown. To date, although the inhibited growth and photosynthesis have been reported in apple grown in replant soil, the direct effects of *Fusarium* infection on apple photosystems are still unclear (Wang et al., 2014; Henfrey et al., 2015).

Recently, a simultaneous measurement of prompt chlorophyll *a* fluorescence (PF), delayed chlorophyll *a* fluorescence (DF), and modulated 820 nm reflection (MR) has been developed, and this technique allows collection and correlation of complementary information for diagnosing PSII and PSI performance and investigating characterization of photosynthetic electron transport (Strasser et al., 2010; Goltsev et al., 2012; Oukarroum et al., 2013, 2016a; Gao et al., 2014). In this study, we aimed to explore whether *F. solani* could induce water deficit stress in apple seedlings, and then investigate the responses of PSII and PSI performance and their interaction through simultaneously detecting PF, DF, and MR. According to the studies on drought stress, PSII reaction center is commonly more sensitive than PSI reaction center in crops except the contrary result in some trees with tremendous leaf water deficit after long severe drought stress, and the limitation of electron transport from PSII can retard ROS production by preventing over-reduction of PSI acceptor side (Goltsev et al., 2012; Huang et al., 2013; Campos et al., 2014; Zivcak et al., 2014b; Zhang S.B. et al., 2016). Therefore, we hypothesized that infection of *F. solani* could depress PSII and PSI performance and destroy their coordination by inducing foliage wilting in apple seedlings, and greater vulnerability of PSII could help alleviate the detrimental effects on PSI. This study can deepen the knowledge of pathological characterization in apple with *Fusarium* infection and assist in revealing the source of apple replant disease in Yantai.

## Materials and Methods

### Plant Material and Inoculation

Apple (*Malus hupehensis* Rehd.) seeds were stored in sand at 4°C for 30 days to sprout, and the sprouts were sown in nursery plates filled with sand. The sprouts were watered with Hoagland solution (pH 5.7) and placed in artificial climatic chambers (Huier, China). In this study, the photon flux density was approximately 200 µmol m^-2^ s^-1^ (12 h per day from 07:00 to 19:00) in the chambers, and day/night temperature and humidity were controlled at 25/18°C and 65%. After 2 months, uniform seedlings which developed six leaves were selected for inoculation.

Rhizosphere soil was sampled in replant apple orchards in Yantai (37°11′N, 121°11′E), and these replant orchards were reconstructed from 30-year-old orchards. In these replant orchards, apple trees were 5-year-old in 2016 and exhibited obvious replant disease with reduced growth and yield. Fungi were isolated from the rhizosphere soil and purified by plate culture with Martin medium. Genome DNA of the fungi was extracted through CTAB method, and PCRs were carried out with ITS1 and ITS4 as the primers. PCR products were used for sequencing, and fungi were identified by blasting the sequences in NCBI. The results suggested that many fungi belonged to *F. solani*, as their sequence identity with reported *F. solani* (accession: MF467479.1) reached 100%. A strain of *F. solani* exhibiting rapid growth in potato dextrose agar medium was designated as *F. solani* A07, and used for apple infection experiment in our study. It has been preserved as a patented strain in China General Microbiological Culture Collection Center (CGMCC), and the preservation number is CGMCC No. 13187.

*Fusarium solani* A07 was cultured on a potato dextrose agar medium at 28°C in the dark for 7 days, and discs of fungus-containing agar was excised from the culture margins and inoculated into Erlenmeyer flasks containing potato dextrose agar medium. The flasks were incubated for 7 days at 28°C with rotary shaking at 180 rpm, and then mycelial fragments were removed by filtering through sterile cheesecloth to obtain conidial suspension. The conidial suspension was diluted to 4 × 10^6^ spore ml^-1^ using sterile water, and then the roots of apple seedlings were immersed in the conidial suspension for 2 h for inoculation, while the roots of control seedlings were immersed in sterile water for 2 h. After inoculation, the seedlings were planted in plastic pots filled with sterilized soil and placed in artificial climatic chambers under the same culture conditions as those before inoculation. Five replicate seedlings were respectively selected from inoculated and control groups for each experiment, and the newly expanded leaves were used for the measurements.

### Measurements of Leaf Biomass, Proline, and Relative Water Contents

Fresh leaves were harvested and weighed (fresh weight, FW). Subsequently, they were immersed in distilled water for 4 h at room temperature to determine saturated fresh weight (SW). At last, the leaves were dried completely in an oven at 70°C and weighed (dry weight, DW). Relative water content (RWC) was calculated as: RWC = (FW - DW)/(SW - DW) × 100%.

Proline content was measured by using ninhydrin coloration method (Yan et al., 2012b). Dry plant powder (0.1 g) was homogenized with 5 ml of sulfosalicylic acid (3% w/v). After centrifugation, the supernatant (2 ml) was incubated with glacial acetic acid (2 ml) and ninhydrin reagent (3 ml) at 100°C for 40 min. After cooling, 5 ml of toluene was added to the mixture, and then the absorbance of chromophore-containing toluene was recorded at 520 nm. A standard curve was plotted by using known concentrations of proline to determine leaf proline content. The absorbance was measured by using a UV-1800 spectrophotometer (Shimadzu, Japan).

### Simultaneous Measurements of PF, DF, and MR Transients

The measurements were made by using a multifunctional plant efficiency analyzer (MPEA, Hansatech, United Kingdom), and the operating mechanism of this instrument has been elucidated in detail (Strasser et al., 2010). This powerful instrument has been widely used for investigating PSII and PSI performance and photosynthetic electron transport process in recent years (Strasser et al., 2010; Goltsev et al., 2012; Oukarroum et al., 2013, 2016a; Gao et al., 2014; Yan et al., 2015). The leaves were adapted in dark for 30 min before measurement. Thereafter, the leaves were illuminated with 1 s red light (627 nm, 5000 µmol photons m^-2^ s^-1^) and subsequently with 10 s far red light (735 nm, 200 µmol photons m^-2^ s^-1^). PF, DF, and MR transients were simultaneously recorded in the first 1 s illumination with red light, and MR data were also detected in the following 10 s illumination with far red.

Oxidation of PSI reaction center is known to cause an increase in absorbance in 800–850 nm range, and monitoring 820 nm reflection is a very convenient way to follow the redox state of PSI reaction center under continuous light (Schansker et al., 2003). A modulated light source built in the measuring head of MPEA allowed the measurement of the kinetics of light-induced absorption changes at 820 nm (Strasser et al., 2010). MR_0_ is the value at onset of red light illumination, when PSI reaction center is in reduced form (0.7 ms, the first reliable MR measurement; Strasser et al., 2010). After far red illumination for 10 s, PSI reaction center was completely oxidized, and the relative difference of 820 nm reflection between the maximal oxidized and reduced PSI reaction center (ΔMR/MR_0_) was used as an index of the maximal photochemical capacity of PSI (Schansker et al., 2003; Yan et al., 2012a).

Prompt chlorophyll *a* fluorescence transients were quantified according to JIP test by using the following original data: (1) fluorescence intensity at 20 s (*F*_o_, when all reaction centers of PSII are open); (2) the maximal fluorescence intensity (*F*_m_, when all reaction centers of PSII are closed); and (3) fluorescence intensities at 300 µs (K step), 2 ms (J step), and 30 ms (I step). Using these original data, some parameters can be calculated for quantifying photosynthetic electron transport (Strasser et al., 2010). The parameters and formulae are listed in **Table 1**.

**Table 1:** Formulae and terms used in the analysis of OJIP fluorescence transient.

**Data extracted from the recorded fluorescence transient OJIP**	
*F*_t_	Fluorescence intensity at time *t* after onset of actinic illumination
*F*_o_ = *F*_20 μs_	Minimal recorded fluorescence intensity
*F*_k_ = *F*_300 μs_	Fluorescence intensity at 300 µs
*F*_J_ = *F*_2 ms_	Fluorescence intensity at the J step
*F*_I_ = *F*_30 ms_	Fluorescence intensity at the I step
*F*_m_	Maximal fluorescence intensity
**Fluorescence parameters derived from the extracted data**	
*V*_t_ = (*F*_t_ -*F*_o_)/(*F*_m_ - *F*_o_)	Relative variable fluorescence at time *t*
*V*_k_ = (*F*_k_ - ctron transport process in recent years*F*_o_)/(*F*_m_ -*F*_o_)	Relative variable fluorescence intensity at K step
*V*_J_ = (*F*_J_ - *F*_o_)/(*F*_m_ -*F*_o_)	Relative variable fluorescence intensity at J step
*V*_I_ = (*F*_I_ - *F*_o_)/(*F*_m_ -*F*_o_)	Relative variable fluorescence intensity at I step
*W*_k_ = (*F*_k_ - *F*_o_)/(*F*_J_ -*F*_o_)	Ratio of relative variable fluorescence at K step to that at J step
**Biological parameters derived from the fluorescence parameters**	
*F*_v_*/F*_m_ = 1 -*F*_o_/*F*_m_	Maximum quantum yield for primary photochemistry
ETo/TRo = 1 -*V*_J_	Probability that an electron moves further than Q_A_
REo/ETo = (1 - *V*_I_)/(1 -*V*_J_)	Probability with which an electron from the intersystem electron carriers is transferred to reduce end electron acceptors at the PSI acceptor side
ETo/ABS = (*F*_m_ - *F*_J_)/*F*_m_	Quantum yield for electron transport
REo/ABS = (*F*_m_ - *F*_I_)/*F*_m_	Quantum yield for reduction of end electron acceptors of PSI
RC/ABS = (*V*_J_/4*V*_k_) *F*_v_*/F*_m_	Q_A_ reducing reaction centers per PSII antenna chlorophyll
PI_total_ = RC/ABS⋅[TRo/(ABS - TRo)]⋅ [ETo/(TRo - ETo)]⋅[REo/(ETo - REo)]	Total performance index

*Q_*A*_, primary quinone; PSI, photosystem I; PSII, photosystem II.*

All redox reactions of the photosynthetic electron transport are reversible. The accumulation of electrons in the electron transport chain between PSII and PSI leads to back electron transfer and charge recombination in PSII reaction center, resulting in re-excitation of reaction center and repopulation of excited chlorophyll state of PSII antenna. The light emission from repopulated excited chlorophyll is delayed fluorescence (Goltsev et al., 2009). In order to exclude PF interference under the light, simultaneous measurement of DF requires alteration of light and dark intervals, and DF decay signals are continuously recorded in dark intervals (Goltsev et al., 2009; Strasser et al., 2010). In this study, DF signals in microsecond and sub-millisecond domains were collected, respectively, at 20 and 200 µs after turning off actinic light for constructing DF transients.

### Statistical Analysis

One-way ANOVAs were carried out by using SPSS 16.0 (SPSS Inc., Chicago, IL, United States) for all sets of data. The values presented are the mean of samples collected from five replicate seedlings in inoculated and control groups. The comparisons of means were determined through least significant difference test, and the differences were considered significant at *P* < 0.05.

## Results

### Leaf Biomass, Relative Water Content, and Proline Content

After 30 days of infection, FW, DW, and RWC in the leaves were tremendously decreased by 55.3, 47.1, and 55.7%, and leaf proline content was remarkably elevated by 7.8 folds (**Table 2**).

**Table 2:** Fresh weight (FW), dry weight (DW), relative water content, and proline content in the leaves of apple seedlings after 30 days of infection with *Fusarium solani*.

Treatments	FW per leaf (g)	DW per leaf (g)	Relative water content (%)	Proline content (mg g^-1^ DW)
Control plants	0.085 ± 0.005a	0.034 ± 0.007a	88 ± 1.5a	0.38 ± 0.05a
Infected plants	0.038 ± 0.009b	0.018 ± 0.005b	39 ± 2.1b	3.35 ± 0.08b

*Data in the table indicate the mean of five replicates (±SD). Within each column, mean values followed by the different letters are significantly different at *P* < 0.05.*

### Total Performance Index and the Maximal Photochemical Efficiency of PSII and PSI

**FIGURE 1:**
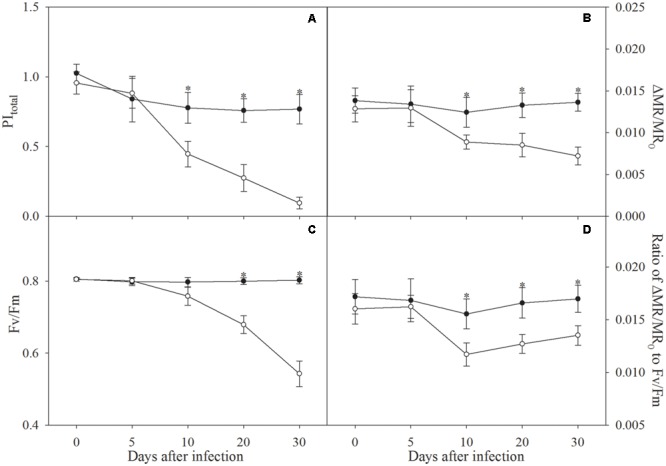
Total performance index (PI_total_, **A**), the maximal photochemical efficiency of PSI (ΔMR/MR_0_, **B**), the maximal photochemical efficiency of PSII (*F*_v_/*F*_m_, **C**), and ratio of ΔMR/MR_0_ to *F*_v_/*F*_m_ **(D)** in control plants (closed symbols) and plants infected by *Fusarium solani* (open symbols). Data in the figure indicate the mean of five replicates (±SD), and asterisk indicates significant difference at *P* < 0.05.

The definition of PI_total_ and *F*_v_/*F*_m_ is listed in **Table 1**. During the first 5 days of infection, PI_total_, *F*_v_/*F*_m_ and ΔMR/MR_0_ were not affected (**Figures 1A–C**). After 10 days of infection, PI_total_, ΔMR/MR_0_ and the ratio of ΔMR/MR_0_ to *F*_v_/*F*_m_ were significantly decreased in line with slightly lowered *F*_v_/*F*_m_, however, the decrease in *F*_v_/*F*_m_ appeared to be faster than ΔMR/MR_0_ in the following days (**Figure 1**). From infection for 10 days to 30 days, decrease in the ratio of ΔMR/MR_0_ to *F*_v_/*F*_m_ was curtailed from 24.5 to 20.6% (**Figure 1D**), which verified the faster decrease in *F*_v_/*F*_m_ than ΔMR/MR_0_.

### Transients of PF, DF, and MR

**FIGURE 2:**
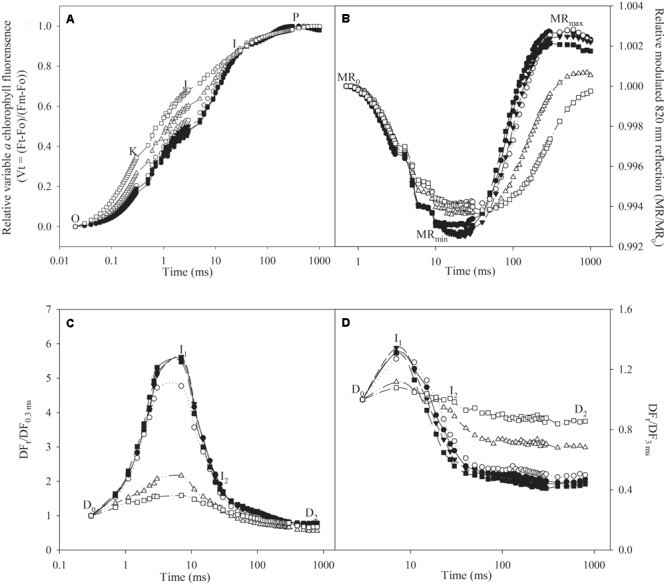
Transients of prompt chlorophyll *a* fluorescence **(A)**, modulated 820 nm reflection **(B)**, and delayed chlorophyll *a* fluorescence in microsecond domain **(C)**, and sub-millisecond domain **(D)** in control plants (closed symbols) and plants infected by *Fusarium solani* (open symbols) for 10 days (circles), 20 days (triangles), and 30 days (squares). O, K, J, I, and P indicate the specific steps in chlorophyll *a* fluorescence transient. MR_0_ is the value of modulated 820 nm reflection at the onset of red light illumination (0.7 ms, the first reliable MR measurement). MR_min_ and MR_max_ indicate the maximal point during PSI oxidation and the maximal point during PSI re-reduction, respectively. D_0_, I_1_, I_2_, and D_2_ indicate initial point, the first (7 ms) and second (50 ms) maximal peaks and minimum point in delayed chlorophyll *a* fluorescence curves. DF_0.3 ms_ is the initial microsecond delayed fluorescence signal at 0.3 ms, and DF_3 ms_ is the initial sub-millisecond delayed fluorescence signal at 3 ms. The signals were plotted on a logarithmic time scale. Each curve is the average of five replicates.

No obvious change occurred in PF transient except for a slight elevation of J step after 10 days of infection (**Figure 2A**). When the infection was prolonged to 20 and 30 days, J step was significantly elevated, and a clear K step appeared in the transients (**Figure 2A**).

As shown in **Figure 2B**, MR signals are presented by MR/MR_0_ ratio, where MR_0_ is the value at onset of actinic illumination (at 0.7 ms). Decrease in MR/MR_0_ from MR_0_ to the minimal value (MR_min_) reflects PSI oxidation process. MR_min_ is a transitory steady state with equal oxidation and re-reduction rate of PSI reaction center. Subsequently, increase of MR/MR_0_ from MR_min_ to the maximal level (MR_max_) indicates PSI re-reduction. After 10 days of infection, MR transient was obviously affected, as PSI oxidation process was shortened. Besides shortened PSI oxidation process, PSI re-reduction was also slowed when the infection was prolonged to 20 days (**Figure 2B**).

Delayed chlorophyll *a* fluorescence signals include microsecond and sub-millisecond components, and they are presented by DF_t_/DF_0.3 ms_ and DF_t_/DF_3 ms_, respectively. I_1_ (7 ms) and I_2_ (50 ms) are two peaks in DF transients (**Figures 2C,D**). I_1_ peak appears due to the accumulation of S_3_Z^+^P680Q_A_^-^ state, because S_3_ state exhibits the highest DF signal during the S-state cycle of oxygen evolving complex (OEC), and I_2_ is related to the prolonged reopening of PSII reaction centers by electron transfer from reduced Q_B_ to plastoquinone (PQ; Goltsev et al., 2009). DF_0.3 ms_ is the initial microsecond delayed fluorescence signal at 0.3 ms, and DF_3 ms_ is the initial sub-millisecond delayed fluorescence signal at 3 ms. The microsecond and sub-millisecond DF transients were not obviously affected after 10 days of infection except for a slight decrease of I_1_ peak in microsecond DF transient (**Figure 2C**). After 20 and 30 days of infection, I_1_ and I_2_ peaks in microsecond DF transients were significantly lowered, whereas significant elevation of I_2_ peak was noted in sub-millisecond DF transients (**Figures 2C,D**).

### Characterization of Photosynthetic Electron Transport According to JIP Test

**FIGURE 3:**
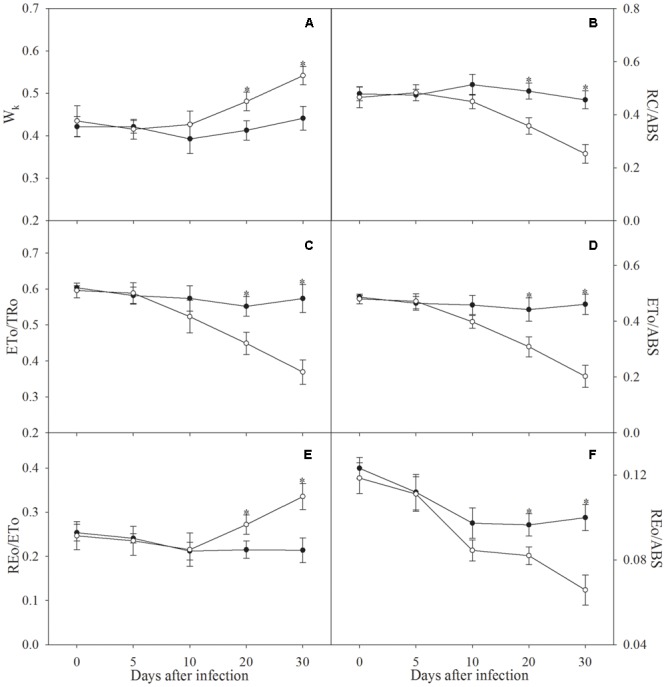
*W*_k_ **(A)**, RC/ABS **(B)**, ETo/TRo **(C)**, ETo/ABS **(D)**, REo/ETo **(E)**, and REo/ABS **(F)** in control plants (closed symbols) and plants infected by *Fusarium solani* (open symbols). The definition for these parameters is in **Table 1**. Data in the figure indicate the mean of five replicates (±SD), and asterisk indicates significant difference at *P* < 0.05.

The definition of *W*_k_, RC/ABS, ETo/TRo, ETo/ABS, REo/ETo, and REo/ABS is listed in **Table 1**. Significant increase in *W*_k_ and REo/ETo did not occur in apple seedlings until the infection was prolonged to 20 days (**Figures 3A,E**). Decrease in RC/ABS, ETo/TRo, ETo/ABS, and REo/ABS was noted after 10 days of infection, and the decrease became significant after 20 days of infection (**Figures 3B–D,F**).

### PSI Oxidation and Re-reduction Amplitude and I_2_/I_1_

**FIGURE 4:**
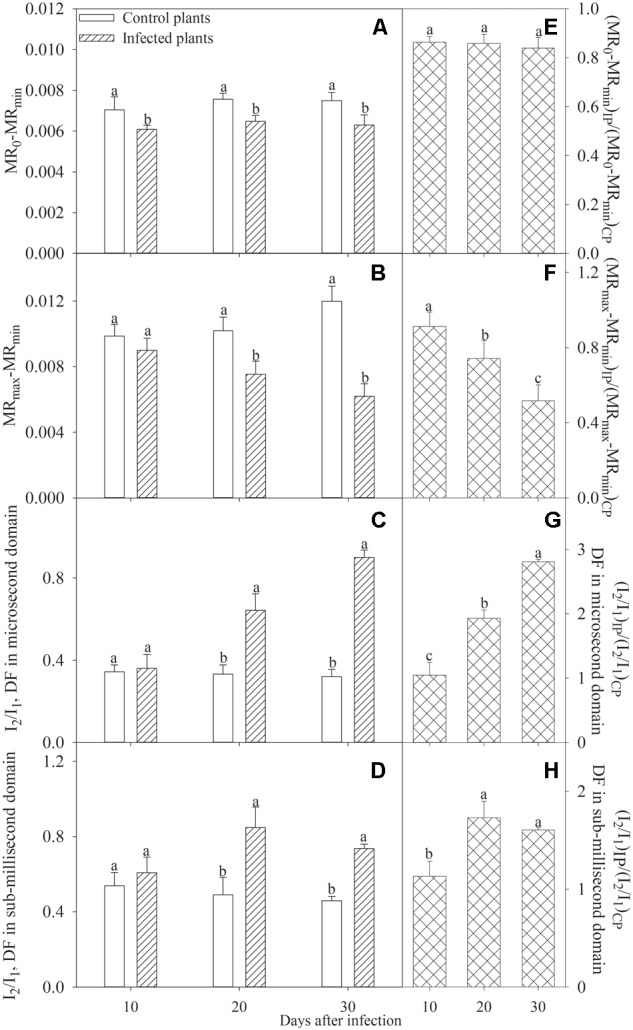
MR_0_–MR_min_ **(A)**, MR_max_–MR_min_ **(B)**, I_2_/I_1_ in microsecond **(C)**, and sub-millisecond **(D)** domains, and ratios of these parameters between plants infected by *Fusarium solani* and control plants **(E–H)**. Data in the figure indicate the mean of five replicates (±SD). Different letters on error bars indicate significant difference at *P* < 0.05.

Significant decrease in MR_0_–MR_min_ and MR_max_–MR_min_ was observed, respectively, after 10 and 20 days of infection (**Figures 4A,B**). Since infection for 10 days, the ratio of MR_0_–MR_min_ in infected seedlings to control seedlings did not change, whereas the ratio of MR_max_–MR_min_ in infected seedlings to control seedlings was significantly lowered (**Figures 4E,F**). I_2_/I_1_ in both microsecond and sub-millisecond domains was significantly increased after 20 days of infection (**Figures 4C,D**). The ratio of I_2_/I_1_ in infected seedlings to control seedlings was significantly higher after infection for 20 and 30 days than the value after 10 days of infection (**Figures 4G,H**).

## Discussion

*Fusarium* fungi can impede water transport through xylem by inducing vessel plugging and then bring about pathological foliage wilting in plants (Wang et al., 2015). Accordingly, large decrease in leaf RWC suggested that *F. solani* induced pathological wilting in apple seedlings, and thus, the infected seedlings were subjected to severe water deficit stress. This result was further verified by significant increase in leaf proline content (**Table 2**), because proline is a crucial osmolyte in plant tissues for resisting water deficit (Ashraf and Foolad, 2007; Yan et al., 2012b). Thus, as with other plants, leaf biomass was reduced in apple seedlings by *F. solani* (**Table 2**; Wang et al., 2015; Dong et al., 2016; Yang et al., 2016).

Performance index can sensitively reflect photosynthetic apparatus function (Zivcak et al., 2008, 2014c), and significant decrease in PI_total_ after 10 days of infection indicated that photosynthetic apparatus was injured due to pathological foliage wilting (**Figure 1A**). PSI reaction center exhibited higher susceptibility than PSII reaction center due to significant decrease of ΔMR/MR_0_ with slight change in *F*_v_/*F*_m_ after 10 days of infection (**Figures 1B,C**), and the greater damage on PSI reaction center was also corroborated by significant decrease in ratio of ΔMR/MR_0_ to *F*_v_/*F*_m_ since infection for 10 days (**Figure 1D**). However, PSI commonly has stronger drought tolerance than PSII in crops (Goltsev et al., 2012; Zivcak et al., 2014b; Zhang S.B. et al., 2016). In our opinion, this common finding derives from crops upon moderate or mild drought stress, and may not appear in apple under severe water deficit stress with tremendous leaf water loss. Huang et al. (2013) also reported that severe drought stress led to greater PSI damage compared with PSII in trees with large leaf water loss, and proposed that PSI sensitivity resulted from its slower recovery mainly due to inhibited cyclic electron flow around PSI. As a traditional viewpoint, PSII photoinhibition can protect PSI by restricting electron flow to PSI acceptor side for reducing ROS production (Sonoike, 2011; Zivcak et al., 2014a; Oukarroum et al., 2015; Zhang Z.S. et al., 2016). Consistently, damage of PSI reaction center was actually alleviated by PSII photoinhibition in some extent, indicated by slower decrease of ΔMR/MR_0_ than *F*_v_/*F*_m_ and curtailed decrease in ratio of ΔMR/MR_0_ to *F*_v_/*F*_m_ since infection for 10 days (**Figures 1B–D**). However, the positive role of PSII photoinhibition was limited for mitigating PSI damage, because of the great vulnerability of PSI to pathological wilting in apple seedlings infected by *F. solani*.

In line with decreased amount of active PSII reaction centers (**Figure 3B**), photosynthetic electron transport beyond Q_A_ was inhibited after 20 days of infection according to significant decrease of ETo/TRo and elevated J step (**Figures 2A, 3C**). Thus, the injury on PSII acceptors appeared (Schansker et al., 2005; Yan et al., 2013b). In contrast, greater injury occurred in OEC at PSII donor side, because a clear K step with significant increase of *W*_k_ appeared under inhibited electron transport beyond Q_A_ (**Figures 2A, 3A**; Oukarroum et al., 2013, 2016b; Gao et al., 2014). These results indicated that leaf pathological wilting brought about deleterious effects on whole PSII components including reaction center, donor and acceptor sides. As a result, electron flow from PSII to PSI was greatly restricted (**Figures 3D,F**). Limited electron donation from PSII can promote PSI oxidation by retarding its re-reduction (Strasser et al., 2010; Goltsev et al., 2012; Yan et al., 2013a,b; Dabrowski et al., 2017), however, PSI oxidation after 10 days of infection was obviously inhibited (**Figures 2B, 4A**). Therefore, we inferred that inhibition on PSI photochemical capacity was greater compared with limited electron donation from PSII (Yan et al., 2015). This result conformed to above analysis about greater susceptibility of PSI reaction center, and unchanged ratio of PSI oxidation amplitude between infected plants and control plants might associated with the protective effect on PSI by declined PSII performance (**Figure 4E**). I step represents the kinetic bottleneck of electron transport chain due to limitation of PQ re-oxidation (Schansker et al., 2005). Notably, slight change of I step and significant increase in REo/ETo after 20 days of infection indicated that PQ re-oxidation was not greatly affected, and the limitation on electron flow from PQ to PSI acceptors was lower than electron flux beyond Q_A_ to PQ (**Figures 2A, 3E**). The elevated electron transport efficiency beyond PQ was also reported in *Haberlea rhodopensis* and *Phaseolus vulgaris* under water deficit stress (Strasser et al., 2010; Goltsev et al., 2012), however, this result seemed contradictory to damaged PSI reaction center. Notably, Zivcak et al. (2015) suggested that IP amplitude which also could reflect PQ re-oxidation efficiency depended on not only the activation of PSI reaction center but also electron transport capacity at PSI acceptor side. Thus, we supposed that the negative effect of damaged PSI reaction center on PQ re-oxidation could be compensated, because similar to declined PSII performance, inactivation of PSI reaction center might protect PSI acceptors by reducing ROS production and consequently alleviated the reduction of electron transport efficiency beyond PQ. The positive role of inactivated PSI reaction center has been proposed recently and may be a specific response in apple to *Fusarium*-induced water deficit stress (Tiwari et al., 2016). After 20 days of infection, electron flow from PSII to PSI was blocked so seriously that PSI re-reduction could not be accomplished normally, leading to disconnection between PSII and PSI (**Figures 2B, 4B**). In parallel with increased *W*_k_ and decreased RC/ABS and ETo/TRo, the gradually decreased ratio of PSI re-reduction amplitude between infected plants and control plants also reflected aggravation of PSII damage during prolonged infection after 10 days (**Figures 3A–C, 4F**). In a word, susceptibility of PSI reaction center was confirmed in apple seedlings infected by *F. solani* through analyzing MR transients, and the disconnection between PSII and PSI was also apparently observed in these transients.

Delayed chlorophyll *a* fluorescence in microsecond and sub-millisecond domains is mostly related to Z^+^Q_A_^-^ state of PS II (Goltsev et al., 2009). DF microsecond component is dominated by redox reactions at PSII donor side and can be affected by electron transport at PSII acceptor side, whereas DF sub-millisecond component which associates with activated PS II reaction center mainly depends on redox state of PSII acceptors (Goltsev et al., 2009). The occurrence of I_1_ peak mainly results from accumulation of S_3_Z^+^P680Q_A_^-^ state, as S_3_ state exhibits the highest DF signal during the S-state cycle of OEC (Buchta et al., 2007; Schansker et al., 2011), and particularly, Oukarroum et al. (2016a) illustrated the relation between OEC injury and DF change. Thus, in accordance with elevated K step, remarkably lowered I_1_ in microsecond domain after 20 days infection also reflected the damage of OEC at PSII donor side (**Figures 2A,C**). I_1_–I_2_–D_2_ phase correlates with reduction process of PQ pool, and I_2_ is probably related to the prolonged reopening of PSII reaction centers by electron transfer from reduced Q_B_ to PQ before full reduction of PQ pool (Goltsev et al., 2009; Strasser et al., 2010; Kalaji et al., 2012). Limited electron transport beyond Q_A_ was beneficial to PSII reopening by inhibiting PQ reduction and could mitigate the decrease of microsecond DF, leading to significant increase of I_2_/I_1_ with less decrease in I_2_ than I_1_ after 20 days infection (**Figures 2C, 4C**). Thus, in agreement with the finding of Oukarroum et al. (2013), lowered I_2_ with increased I_2_/I_1_ in microsecond domain verified greater damage of PSII donor side than acceptor side, and the damage of PSII donor became more and more severe than acceptor side since infection for 10 days (**Figure 4G**). In contrast, significant increase of I_2_ and I_2_/I_1_ in sub-millisecond domain depended on greater PQ re-oxidation than PQ reduction after 20 days infection (**Figures 2D, 4D**), and in other words, electron transport beyond PQ was less affected than electron donation from PSII to PQ, which was consistent with the elevated REo/ETo (**Figure 3E**). In accordance with MR transients, the disconnection of PSII and PSI due to remarkably lowered electron donation from PSII was also reflected by the increased ratio of I_2_/I_1_ in sub-millisecond domain between infected seedlings and control seedlings as well as the elevated DF transients from I_2_ to D_2_ after 20 days infection (**Figures 2B,D, 4H**). Obviously, the data from DF, PF and MR can be validated reciprocally.

In agreement with the hypothesis, infection of *F. solani* depressed PSII and PSI performance and destroyed their coordination in apple seedlings by inducing pathological foliage wilting. PSI was more susceptible to infection of *F. solani* than PSII, although depression of PSII performance aided in ameliorating PSI injury to some extent.

## Author Contributions

KY designed the experiment, performed the data analysis, and wrote the manuscript. GH and CR participated in the experiment design and polished the language. SZ reviewed the manuscript and proposed some critical suggestions. XW and TB participated in the experiment and data analysis.

## Conflict of Interest Statement

The authors declare that the research was conducted in the absence of any commercial or financial relationships that could be construed as a potential conflict of interest.

## Abbreviations

∆MR/MR_0_: the maximal photochemical capacity of PSI
ETo/ABS: quantum yield for electron transport
ETo/TRo: probability that an electron moves further than primary acceptor of PSII
*F*_v_/*F*_m_: the maximal quantum yield of PSII
PI_total_: total performance index
PSI: photosystem I
PSII: photosystem II
Q_A_: primary quinone
RC/ABS: primary quinone reducing reaction centers per PSII antenna chlorophyll
REo/ABS: quantum yield for reduction of end electron acceptors of PSI
REo/ETo: probability with which an electron from the intersystem electron carriers is transferred to reduce end electron acceptors at the PSI acceptor side
*W*_k_: ratio of relative variable fluorescence at 300 µs to that at J step
